# The Effect of the Number of Adjuvant Chemotherapy Cycles Following Cancer Surgery on Taste Alteration, Energy Intake, and Life Quality: 6-Month Follow-Up Study

**DOI:** 10.3390/life15071031

**Published:** 2025-06-28

**Authors:** Can Selim Yilmaz, Hilal Caliskan İzgi, Perim Fatma Türker

**Affiliations:** 1 Department of Nutrition and Dietetic, Baskent University, Ankara 06790, Türkiye; csyilmaz@baskent.edu.tr (C.S.Y.); hcaliskan@baskent.edu.tr (H.C.İ.); 2 Department of Nutrition and Dietetics, Acibadem Mehmet Ali Aydinlar University, Istanbul 34638, Türkiye

**Keywords:** adjuvant chemotherapy, taste alteration, life quality, energy intake, malnutrition

## Abstract

This study aimed to investigate the effect of the number of adjuvant chemotherapy cycles on taste alteration, energy intake, and life quality. This study was conducted with 87 adult patients with newly diagnosed early-stage cancer, treated by surgery and receiving adjuvant chemotherapy (two groups received <4 and ≥4 cycles). Taste alteration was assessed with the Chemotherapy-induced Taste Alteration Scale, and life quality with the Functional Assessment of Cancer Therapy. Patients who received ≥4 cycles showed significantly higher “Decline in Basic Taste”, “Phantogeusia and Parageusia”, and “General Taste Alterations” subscale scores at the end of treatment (d = 0.945, d = 1.200, d = 0.928, respectively; *p* < 0.001) and 3 months later (d = 0.515, d = 0.605, d = 0.985, respectively; *p* < 0.05). Quality of life scale total score and “physical well-being” and “functional well-being” subscale scores were significantly lower in patients received ≥4 cycle at the end of treatment (d = 0.590, d = 0.500, d = 0.621, respectively; *p* < 0.05) and 3 months later (d = 0.500, d = 0.516, d = 0.562, respectively; *p* < 0.05) but improved within 6 months. Significant correlations were found between taste alterations and recent weight loss, affordability of energy requirements, and decreased quality of life. A higher number of adjuvant chemotherapy cycles was associated with greater taste alterations, nutritional challenges and lower quality of life, especially in the early post-treatment. These changes are often transient and tend to return to baseline levels within 3–6 months after treatment.

## 1. Introduction

Adjuvant chemotherapy (ACT) is administered following surgical intervention to reduce mortality and recurrence risk by eradicating residual malignant cells and addressing occult disease [1]. While its therapeutic benefits are well established, ACT is also associated with sensory toxicities, including ototoxicity and peripheral neuropathy, which can be long-lasting or permanent [2,3]. Taste alteration (TA) is a frequently reported side effect, occurring even in the absence of nausea [4]. Reported prevalence rates of TAs range widely from 38% to 84% [5,6,7,8], reflecting variability in definitions, assessment methods, and patient populations, including differences in cancer types and chemotherapy regimens [5,6,7]. TAs typically begin with the initiation of chemotherapy [5,7] and may last from several hours to weeks or even months [9]. However, the precise etiology remains unclear, as taste disturbances may result from altered gustatory function, changes in olfaction or somatosensory perception (e.g., oral dryness), or shifts in hedonic responses such as food preference and appetite—phenomena often colloquially referred to as “taste” by patients and clinicians alike [10].

Although TAs were incorporated into the National Cancer Institute’s Common Toxicity Criteria as early as 1999, research on their biological mechanisms, physiological impacts, and prevalence remains limited. The etiology of TAs in patients undergoing ACT is complex and often not fully understood. Some studies suggest that the malignancy itself may contribute to the development of taste disturbances [11,12,13]. Several chemotherapeutic agents—including folinic acid antagonists, cyclophosphamide, platinum compounds, and taxanes—have been particularly associated with metallic taste sensations [9,14,15]. Proposed mechanisms include direct cytotoxic effects on taste receptor epithelial cells, such as cellular damage or disrupted regeneration, and secondary effects such as chemotherapy-induced mucositis. Additionally, certain cytotoxic agents are capable of crossing the blood–brain barrier and may alter central taste processing by affecting afferent gustatory pathways. Other potential contributors include chemotherapy-induced oxidative stress and systemic metabolic alterations [14].

TAs in oncology populations have been linked to adverse effects on quality of life (QoL), morbidity and mortality due to an association with inadequate energy and nutrient intake, weight loss, malnutrition [16], reduced compliance with treatment regimens [17], reduced immunity [16,18], altered food relationships [19], changed food rituals [20], emotional distress and interference with daily life [21]. Patients’ sense of taste might be affected quantitatively (increased or decreased perception) or qualitatively (distorted perception) [14]. Thus, food often tastes different or even unpleasant, and sometimes eating becomes a more and more aversive activity. The burden from the loss of patients’ ability to enjoy meals and the loss of the associated social involvement can be severe. Taste-related problems, such as food aversions and malnutrition, clearly affect daily living and emotional well-being [9].

In clinical practice, patients seldom report TAs on their own, and physicians often view them as an inevitable side effect. Additionally, medical treatment options for TAs have not been extensively investigated [4]. Furthermore, TAs are not adequately considered in clinical decision-making processes [22].

The existing literature predominantly emphasizes nonsurgical neoadjuvant chemotherapy, with relatively few studies addressing adjuvant therapy for cancer recurrence prevention following surgical intervention. Previous studies in the literature about the number of ACT cycles have mostly focused on survival [23,24]. Research on TA in this context is also limited, with most studies focusing on the duration of chemotherapy administration (e.g., 3.6 months) rather than the number of treatment cycles. However, patients may undergo different numbers of cycles within the same timeframe. Therefore, focusing on the number of cycles administered post-surgery may provide a valuable perspective and help to address the existing gap in the literature. These reasons underscore the necessity for further research on TAs in terms of the number of cycles. Therefore, we conducted a study with the following objectives: (a) to investigate the impact of number of ACT cycles administered on TAs and QoL at the end of ACT treatment and during 6-month follow-up; and (b) to investigate the impact of different ACT cycles administered on weight loss and meeting the energy requirements of participants.

## 2. Materials and Methods

This study was designed as an observational follow-up study (T0: baseline, T1: within 1 week after the end of treatment, T2: 3 months after T1, T3: 6 months after T1). The study was conducted with 95 adult patients with newly diagnosed early-stage cancer, treated by surgery and receiving ACT at a hospital-based oncology clinic in Adana, Turkey, between January 2023 and December 2024. The inclusion criteria were adult patients (≥18 years) and patients receiving ACT following cancer surgery. All participants were citizens of the Republic of Turkey.

Power analysis was performed using G*Power (version 3.1.9.7) with an effect size of 0.3, 85% power, and 0.05 error margin. The results indicated that a minimum sample size of 75 participants would be required. Taking into account the possibility of losses during the study, 95 volunteers were recruited.

In the pre-operative period, all patients were evaluated by a multidisciplinary team consisting of an oncology surgeon, oncology physician, oncology specialist nurse, dietician, and psychologist. The obtained medical, surgical, nutritional, physical, and psychiatric histories of the patients were evaluated for inclusion criteria. Eight participants were excluded from the study because they had a chronic disease known to affect metabolism or appetite and/or were taking medication related to these (1 patients due to irregular hypothyroidism, 3 patients due to antidepressant and antipsychotic use), and did not respond to the scales at follow-up (6 patients). After the exclusions, this study was conducted with 87 adults with similar baseline characteristics.

This study was approved by the Baskent University ethics committee (KA24-72). All participants provided written informed consent before enrollment in the study. All procedures performed in studies involving human participants were in accordance with the ethical standards of the institutional and/or national research committee and with the 1964 Helsinki declaration and its later amendments or comparable ethical standards.

The existing literature does not specify a cut-off point for evaluating the impact of the number of adjuvant or neoadjuvant chemotherapy cycles administered. To address this gap, the present study employed a statistically determined cut-off. Participants (n = 87) were divided into two groups based on the median number of ACT cycles received. The median was calculated as 4.12 ACT cycles. Accordingly, individuals who received fewer than 4 cycles were classified as the lower-ACT group (n = 46), while those who received 4 or more cycles were classified as the higher-ACT group (n = 41). This median-based dichotomization is a widely accepted method in clinical research, as it enables balanced group comparisons and minimizes bias associated with arbitrary threshold selection.

Cancer surgeries were performed at a hospital-based oncology clinic in Adana, Turkey. None of the patients had overt distant metastases at the time of inclusion. The participants did not receive radiation to the head and upper neck regions, and had no history of previous cancer. In this study, patients who underwent cancer surgery received antracycline and taxane-based therapy (i.e., paclitaxel, docetaxel, or nab-paclitaxel) as an ACT procedure. Antracycline and taxane-based regimen was administered as monotherapy or combination therapy as deemed appropriate by the oncologist.

### 2.1. Data Collection Process

An interviewer-administered questionnaire was developed to assess differences in taste perception and quality of life among patients undergoing adjuvant chemotherapy. In addition to standardized measures, the questionnaire collected information on patient characteristics, cancer type, and the number of chemotherapy cycles received.

### 2.2. Taste Alteration

TA was assessed with the Chemotherapy-induced Taste Alteration Scale (CiTAS). CiTAS, which is a scale with 18 items and 4 subscales, and was developed by Kano and Kanda in 2013 [25,26]. CiTAS is a 5-point Likert-type scale. ‘Decline in Basic Taste’ subscale: The condition of sensing the bitter, sweet, salty, sour, and umami taste by individuals is assessed. ‘Discomfort’ subscale: The relationship between TAs and nausea-vomiting, experiencing alterations in the sense of smell, having difficulty eating hot/oily/meat, and reduced appetite is assessed. ‘Phantogeusia and Parageusia’ subscale: The condition of individuals based on their experiences of Phantogeusia and Parageusia is assessed. ‘General taste alterations’ subscale: The condition of individuals regarding their experiences of ageusia, cacogeusia, and hypogeusia is assessed. For the assessment of the scale, scores received from each subscale are evaluated rather than the total score received from the entire scale. The subscale scores are obtained by dividing the number of items by the sum of the scores of those items. The maximum score is 5 points, whereas the minimum score is 1 point that can be received from subscales. The increase in the score shows that the intensity of TAs has increased [25].

### 2.3. Quality of Life

QoL was assessed with the Functional Assessment of Cancer Therapy (FACT-G). The FACT-G is a validated questionnaire used to assess QoL in people with cancer [27,28]. The FACT-G is a 27-item general cancer QoL measure: seven items for physical well-being (PWB), seven for social and family well-being (SWB), six for emotional well-being (EWB), and seven for functional well-being (FWB). Each item or question on the FACT-G has response choices ranging from 0 (not at all) to 4 (very much). The total score ranges from 0 to 108 (from 0 to 28 for PWB, SWB, and FWB, while from 0 to 24 for EWB), and a higher score indicates better QoL.

### 2.4. Energy Requirement and Energy Intake at T1

The energy requirements of the patients were determined by multiplying the basal metabolic rate (BMR) and physical activity level (PAL). The PAL [29] was determined using the patients’ 24 h activity profiles. The Harris–Benedict [30] equation was used to calculate the BMR. Food consumption of patients has been detected by using a 24 h dietary recall method (3 consecutive days of the week following the end of treatment). Daily food consumption and energy intake have been evaluated using the Nutrition Information System (BeBis 8.1 version) by researchers. Average energy intake was calculated by the researcher by averaging 3-day food records using the Nutrition Information System.

### 2.5. Statistical Analysis

The data obtained in the study were analyzed using the SPSS 25.0 program. While evaluating, descriptive statistics such as mean (X), Standard Deviation (±SD), number (n), and percentage (%) were calculated. The normality of data was evaluated with the Shapiro–Wilk test and the Kolmogorov–Simirnov test. The significance of the difference between the two means was analyzed by the Chi-square test. Repeated measures ANOVA was used to compare the dependent variables of the patients for continuous data with normal distribution. Post hoc tests were conducted with Bonferroni correction. Spearman’s rho test was used to correlate two quantitative variables. The Kruskal–Wallis test was used to compare two or more independent samples. When a Kruskal–Wallis test leads to a significant result, one-way ANOVA: post hoc test (Tamhane’s T2) was used to see which particular groups are significantly different from each other. The characterization of effect size is small (*η*_2_ = 0.01), medium (*η*_2_ = 0.06) and large (*η*_2_ ≥ 0.14) for Eta-squera [31], while for Cohen’s d it is small (d = 0.20), medium (d = 0.50), large (d = 0.80) and very large (d = 1.20) [31]. The level of statistical significance was accepted as a *p*-value of less than 0.05.

## 3. Results

Baseline demographic and clinical characteristics of participants are shown in Table 1. The study sample consisted of 87 patients who received ACT (median age 58.6 years, range 23–71 years), of whom 52.9% (n = 46) received <4 ACT and 47.1% (n = 41) received ≥4 ACT after cancer surgery. The details of BMI, gender distribution, type of cancer, and ACT regimen by the groups formed according to the number of ACT cycles received are shown in Table 1. Median age, mean BMI, gender distribution, number of patients with different types of cancer (except for lymphoma), and received the ACT regimen are similar between the groups.

Table 2 shows recent weight loss (one month before the measurement at T1) and meeting energy requirements of individuals after the end of treatment (at T1) according to the ACT groups. The percentage of patients who experienced severe weight loss was significantly higher in patients who received ≥4 ACT cycles compared to <4 (29.3% and 10.9%, respectively) (*p* < 0.044). There is no significant difference between genders.

In terms of meeting energy requirements, the majority of patients (48.8%) who received ≥4 ACT cycles were hypometabolic, while the majority of patients (61.0%) who received <4 ACT cycles were normometabolic. The percentage of patients who are hypometabolic was significantly higher in patients who received ≥4 ACT cycles compared to <4 (48.8% and 19.5%, respectively) (*p* = 0.029). There is no significant difference between genders.

Following the end of ACT treatment (T1), the CiTAS subscale scores of the participants according to their characteristics are shown in Table 3. There is no significant difference between CiTAS subscale scores in terms of BMI categories. Similarly, the difference between genders is insignificant. The ‘Decline in Basic Taste’ subscale score is significantly higher in the 18–40 age group compared to the 41–50 age group. The ‘Discomfort’ subscale score is significantly higher in patients with stomach cancer and colorectal cancer than in patients with lymphoma cancer. The ‘General taste alterations’ subscale mean scores are significantly higher in patients with breast and colorectal cancer than in patients with lymphoma cancer.

**Figure 1:**
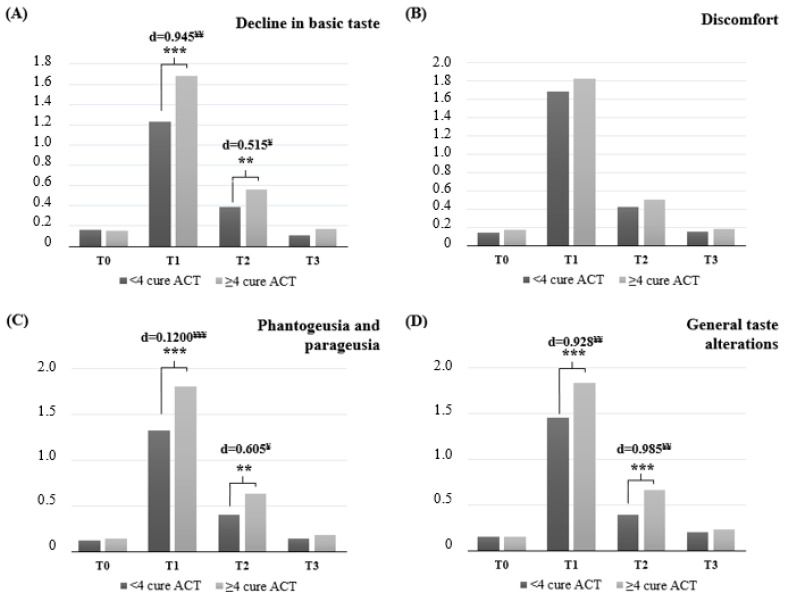
Adjuvant chemotherapy-induced taste alterations detected by CiTAS in participants during treatment and follow-up Repeated measures ANOVA with post hoc Bonferroni (**: *p* < 0.05; ***: *p* < 0.001); T0: Baseline; T1: After treatment; T2: 3 months after treatment; T3: 6 months after treatment; ACT: adjuvant chemotherapy; d: Cohen’s d effect size (¥: medium effects, ¥¥: large effects, ¥¥¥: very large effects); Relationship between periods: T0–T1 *p* < 0.001 and T0–T2 *p* = 0.01 for both patient group. T1–T2 and T1–T3 *p* < 0.001 and T2–T3 *p* = 0.01 in both patient groups. There is no significant relationship between T0 and T3 in both patient groups. There is no significant difference in total and subgroup CiTAS scores between genders at any follow-up time.

Figure 1 presents the trajectory of ACT-induced TAs, as assessed by the CiTAS, throughout treatment and follow-up. All CiTAS subscale scores increased significantly in both treatment groups at the end of chemotherapy (T1) compared to baseline (T0) (*p* < 0.001). At the 3-month follow-up (T2) and 6-month follow-up (T3), subscale scores declined significantly compared to T1 (*p* < 0.001), although scores at T2 remained significantly elevated relative to baseline (T0–T2; *p* = 0.01). A further significant decrease was observed between T2 and T3 (*p* = 0.01). By T3, there were no significant differences between subscale scores and baseline levels in either group. Notably, the subscales ‘Decline in Basic Taste’, ‘Phantogeusia and Parageusia’ and ‘General Taste Alterations’ demonstrated significantly higher scores in patients receiving ≥4 cycles of ACT compared to those receiving <4 cycles, both at T1(mean ± SD: 1.68 ± 0.82, 1.81 ± 1.00, and 1.84 ± 0.94 vs. 1.23 ± 0.59, 1.33 ± 0.78, and 1.46 ± 0.72, respectively; *p* < 0.001 for all) and at T2 (0.56 ± 0.24, 0.63 ± 0.28, and 0.66 ± 0.31 vs. 0.39 ± 0.15, 0.40 ± 0.18, and 0.39 ± 0.16, respectively; *p* < 0.001 for General Taste Alterations and *p* < 0.05 for Decline in Basic Taste and Phantogeusia and Parageusia). Effect sizes at T1 were large to very large (Cohen’s d = 0.945 for Decline in Basic Taste, 0.946 for Phantogeusia and Parageusia, and 0.946 for General Taste Alterations), while at T2 effect sizes ranged from medium to large (d = 0.515, 0.605, and 0.985, respectively). Although all subscale scores remained higher at T3 in the ≥4 cycle group, the differences were not statistically significant. Additionally, no significant differences in total or subscale CiTAS scores were observed between genders at any time point.

Table 4 presents the QoL scores as measured by the FACT-G scale across different time points. At the end of treatment (T1) and at the 3-month follow-up (T2), patients who received ≥4 cycles of ACT reported significantly higher total FACT-G scores as well as PWB and FWB subscale scores compared to those who received <4 cycles of ACT (medium effect size, Cohen’s d = 0.50). Additionally, FWB scores remained significantly higher in the ≥4 cycle group at the 6-month follow-up (T3) (medium effect size, d = 0.50). Among patients receiving <4 cycles of ACT, PWB scores significantly increased at both T2 and T3 compared to baseline (large effect size, *η*^2^ = 0.256). In contrast, patients receiving ≥4 cycles of ACT experienced a significant decline in PWB at T1 compared to baseline, followed by significant improvements at T2 and T3 (large effect size, *η*^2^ = 0.325). For SWB, patients in the ≥4 cycle group showed a significant decrease at T1 relative to baseline, but a significant improvement was observed at T3 compared to T1 (large effect size, *η*^2^ = 0.284). FWB scores in the <4 cycle group significantly improved at both T2 and T3 relative to baseline (large effect size, *η*^2^ = 0.305). Conversely, FWB scores declined significantly at T1 in the ≥4 cycle group but improved at later follow-ups, with a significant increase noted at T3 compared to T1 (large effect size, *η*^2^ = 0.228). The total FACT-G score in the <4 cycle group increased across all follow-up time points relative to baseline, reaching statistical significance at T3 (large effect size, *η*^2^ = 0.368). In the ≥4 cycle group, the total FACT-G score decreased significantly at T1 compared to baseline but subsequently improved, with significant increases at T2 and T3 compared to T1 (large effect size, *η*^2^ = 0.320). No significant differences in total or subscale FACT-G scores were observed between genders at any time point.

The correlations observed at the end of treatment (T1) between CiTAS scores, recent weight loss, energy intake, and QoL are presented in Table 5. A significant positive correlation was found between recent weight loss and the CiTAS subscales “Phantogeusia and Parageusia” and “General Taste Alterations”, indicating that greater taste disturbances were associated with increased weight loss. Additionally, a significant negative correlation was identified between the percentage of energy requirements met and the subscales “Decline in Basic Taste”, “Phantogeusia and Parageusia”, and “General Taste Alterations”, suggesting that more severe taste alterations were linked to reduced nutritional intake. Furthermore, the total FACT-G score showed a significant negative correlation with all CiTAS subscale scores, indicating that greater taste alterations were associated with lower overall QoL. Detailed correlations between the FACT-G subscale scores and CiTAS subscales are provided in Table 5.

## 4. Discussion

TA is a common side effect among patients undergoing ACT [4] and has the potential to adversely affect dietary intake and QoL [32]. A systematic review and meta-analysis of 30 studies involving 15,722 participants reported a pooled prevalence of chemotherapy-induced TAs at 70%, with individual studies reporting a range between 21% and 100%. Identified risk factors include female sex, xerostomia, oral mucositis or ulceration, and receiving two or more chemotherapy cycles [33]. TAs are associated with diminished food enjoyment, reduced appetite, and insufficient nutrient intake, which may contribute to weight loss and malnutrition, thereby complicating cancer treatment and recovery [34,35,36,37]. Furthermore, the severity of TAs has been linked to increased patient discomfort and a heightened risk of malnutrition [34].

Several studies have highlighted the relationship between TAs and nutritional challenges in patients undergoing chemotherapy. Turcott et al. [38] have reported that specific TAs have been associated with reduced appetite and decreased caloric and food intake. Similarly, Sánchez-Lara et al. [39] have found that cancer patients with altered sweet taste detection have consumed significantly fewer calories, proteins, carbohydrates, and zinc, which contributed to inadequate energy intake and weight loss. Al-Amouri et al. [34] have observed that 98.3% of 120 cancer patients have experienced TAs during chemotherapy, with 31.7% identified as being at high risk of malnutrition. These alterations were significantly associated with increased risk of malnutrition, weight loss, and reduced appetite. In a study by Steinbach et al. [40], chemotherapy has been shown to impair olfactory and gustatory functions, leading to diminished appetite, decreased energy intake, and weight loss. Consistent with these findings, our study demonstrated that patients receiving ACT experienced moderate to severe weight loss by the end of treatment (T1), affecting 25% and 36% of those receiving <4 cycles and ≥4 cycles, respectively. Severe weight loss was significantly more prevalent in the group receiving ≥4 ACT cycles (29.3%) compared to those receiving <4 cycles (10.9%). Moreover, 48.8% of patients receiving ≥4 ACT cycles failed to meet their total daily energy requirements, a significantly higher proportion than in the group receiving <4 cycles.

The relationship between BMI and taste alterations in the literature is contradictory. For instance, Pedersini et al. [41], in a study involving breast cancer patients undergoing neoadjuvant chemotherapy and assessed using the CiTAS, have reported no significant association between BMI and TAs. Conversely, other studies have identified lower BMI as a potential risk factor for increased oral discomfort and more pronounced taste disturbances, reflected in higher CiTAS scores [42,43]. In alignment with Pedersini et al.’s findings, the present study also did not reveal a significant difference in TAs across different BMI groups.

The association between BMI and age in relation to TAs remains inconsistent across studies. Some research suggests that older patients are more susceptible to severe or prolonged taste disturbances, as indicated by higher scores on the CiTAS [42]. Conversely, other studies report that younger patients tend to experience greater emotional distress or discomfort associated with taste changes [21]. Kano and Kanda [25], however, have found no significant differences in CiTAS scores across age groups. Consistent with these findings, the present study also observed no significant association between age groups and overall CiTAS scores, with the exception of the “Decline in Basic Taste” subscale, where a significant difference was noted.

In the study, conducted by Al-Amouri and Badrasawi [34] utilizing the CiTAS, female patients receiving chemotherapy have exhibited significantly higher scores on the Phantogeusia and Parageusia subscales. Similarly, Kano and Kanda [25] have reported that females were more likely to report alterations in flavor sensitivity. However, other studies have found no significant association between gender and specific TA subscales [4,44]. In line with these latter findings, the present study did not observe any significant differences between male and female patients in relation to TAs.

In the present study, a significant difference was identified among cancer types in the General Taste Alterations subscale of the CiTAS, with the highest scores reported in patients with colorectal cancer, followed by those with breast cancer. No statistically significant differences were observed across cancer types in the other CiTAS subscales. Similarly, Al-Amouri and Badrasawi [34] have reported a significant variation in the Phantogeusia and Parageusia subscales based on cancer type, with the highest scores noted in patients with breast cancer, followed by those with lymphoma, colorectal, and lung cancers. These findings suggest that the type of cancer may influence specific dimensions of taste alteration experienced during chemotherapy.

The ability of anthracycline- and taxane-based chemotherapy regimens to cross the blood–brain barrier (BBB), even to a limited extent, is a critical factor in understanding their neurotoxic profiles. While these agents are generally restricted by efflux transporters like P-glycoprotein (P-gp), certain modifications and formulations have demonstrated enhanced BBB penetration. For instance, cabazitaxel, a novel taxane, exhibits improved central nervous system penetration compared to its predecessors, owing to its reduced affinity for P-gp [45]. These pharmacokinetic properties have implications for the gustatory cortex, a brain region integral to taste perception. Chemotherapy-induced taste alterations are prevalent among patients receiving these treatments, with studies reporting a high incidence of taste and smell alterations in this population [33]. The underlying mechanisms may involve direct neurotoxic effects, as well as indirect pathways such as systemic inflammation and peripheral nerve damage, which can influence central taste processing [46]. Both regimens are associated with significant taste alterations in cancer patients, as measured by CiTAS. Docetaxel, a taxane, is particularly linked to higher scores in Decline in Basic Taste and General Taste Alterations [47]. Similarly, cyclophosphamide and adriamycin, components of anthracycline-based therapy, are associated with elevated Phantogeusia and Parageusia scores [34]. In addition, although not used by the participants in this study, platinum-based chemotherapeutic agents, such as cisplatin, carboplatin, and oxaliplatin, are also associated with TAs through multiple mechanisms affecting both peripheral taste receptors and central processing pathways [48]. In this study, no significant difference was found between chemotherapy regimens in terms of CiTAS scores regardless of the number of cycles received.

Previous research has demonstrated a positive association between the number of chemotherapy cycles and the severity of TAs [49]. Bernhardson et al. [21] have reported that TAs frequently emerge early in chemotherapy, often within the initial cycles, and tend to intensify with cumulative exposure to chemotherapeutic agents. Similarly, Al-Amouri and Badrasawi [34] have found that an increased number of completed chemotherapy cycles was significantly associated with reductions in basic taste perception, as well as higher scores in Phantogeusia and Parageusia. Boltong et al. [50] have also observed a marked decline in the ability to detect all five basic tastes over time, noting that this decline worsened with an increasing number of chemotherapy cycles. Conversely, some studies have indicated that taste function may deteriorate acutely following the first chemotherapy cycle. For instance, Silva et al. [43] have reported diminished taste perception immediately after the initial cycle, while Celik et al. [51] have found that patients experienced more pronounced taste disturbances at the beginning of treatment compared to later cycles. In a study conducted by Denda et al. [52] involving breast cancer patients undergoing chemotherapy, TAs have been reported in over 50% of participants, with prevalence decreasing to below 10% prior to the subsequent chemotherapy cycle. In the present study, scores across all subscales of the CiTAS significantly increased at the end of treatment (T1) in both groups when compared to baseline. Notably, patients who received ≥4 cycles of ACT exhibited significantly higher scores in the ‘Decline in Basic Taste’, ‘Phantogeusia and Parageusia’, and ‘General Taste Alterations’ subscales at both T1 and the 3-month follow-up (T2), relative to those who received <4 cycles. These findings suggest that a greater number of chemotherapy cycles may exacerbate the severity and persistence of TAs.

Previous research has indicated that TAs associated with chemotherapy are generally transient, with symptoms tending to resolve following the completion of treatment [40,41,50]. A systematic review of 25 studies examining taste function during and after chemotherapy has found that impairments often begin within the first few days of treatment and may persist for up to 6 months post-therapy [35]. Bleumer et al. [53] have reported a significant decline in both olfactory and gustatory functions during chemotherapy; although these functions improved after treatment cessation, baseline levels were not fully restored within 6 to 12 months. In the present study, all subscale scores of the CiTAS demonstrated a significant reduction at both the 3-month and 6-month follow-up assessments compared to scores at the end of treatment (T1), indicating progressive improvement in taste function over time. However, despite this improvement, scores remained significantly elevated at the 3-month follow-up (T2) compared to baseline, indicating that although taste function begins to recover post-treatment, complete restoration may be delayed. By the 6-month follow-up, subscale scores had returned to levels comparable to baseline in both patient groups, indicating that treatment-induced TAs had fully recovered within this period.

Lewandowska et al. [54] have reported that patients who received ≥6 cycles of chemotherapy experienced significantly lower QoL scores, particularly in the domain of physical well-being (PWB), compared to those receiving fewer cycles. Similarly, Dehkordi et al. [55] have found that patients undergoing 3–5 or ≥6 chemotherapy cycles had significantly lower QoL scores than those who received ≤2 cycles. In contrast, Carter et al. [56] have observed no significant changes in total QoL score and PWB, SWB, and FWB subscale scores across treatment cycles when assessed at baseline (cycle 1), after the sixth cycle, and during extended treatment (cycles 7–16). While EWB scores declined after the 12th cycle compared to the 6th, the difference was not significant when compared to baseline. In the present study, patients who received ≥4 cycles of ACT had significantly lower total FACT-G score and PWB and FWB subscales scores of FACT-G at the end of treatment (T1) and at the 3-month follow-up (T2), relative to those receiving <4 cycles. However, by 6 months post-treatment, FWB scores were significantly higher in patients who had received ≥4 cycles, suggesting a recovery in functional well-being over time.

A previously published review by Cheng et al. [57] has indicated that QoL during ACT typically remains stable or improves during follow-up. Mols et al. [58] similarly noted that QoL tends to decline during chemotherapy but generally returns to baseline within one year post-treatment. Other studies have reported a reduction in QoL during chemotherapy, followed by recovery to levels comparable to non-treatment groups within 6–9 months after therapy [59,60]. In a study by Ochoa et al. [61], patients with breast and colon cancer receiving ACT experienced a decline in QoL at the end of treatment, while QoL remained stable among lung cancer patients. In the present study, patients who received <4 cycles of ACT exhibited QoL scores (as measured by the total FACT-G) that remained comparable to baseline (T0) at the end of treatment (T1), with significant improvement observed at the 6-month follow-up (T3). In contrast, patients who received ≥4 cycles experienced a significant decline in QoL at the end of treatment (T1), followed by gradual improvement at 3 (T2) and 6 months (T3) post-treatment. In terms of subscale analysis, PWB, SWB, EWB, and FWB remained similar to baseline (T0) at the end of treatment (T1) in patients receiving <4 cycles, with significant improvements in PWB and FWB observed at 3 months and continued improvement in FWB at 6 months. Among those receiving ≥4 cycles, PWB, SWB, and FWB declined at the end of treatment but improved progressively within 3 to 6 months post-treatment.

Previous studies have demonstrated that TAs are commonly associated with increased fatigue and reduced appetite, contributing to decreased food intake and subsequent nutritional deficiencies. These factors have been linked to both a decline in QoL and an elevated risk of malnutrition among cancer patients undergoing chemotherapy [32,42]. Conversely, Pedersini et al. [41] found that TAs have not significantly affected dietary habits or body weight in patients with early-stage breast cancer receiving ACT; most participants maintained stable weight and adhered to dietary recommendations, regardless of taste changes. In contrast, Al-Amouri and Badrasawi [34] have reported an association between TAs and malnutrition in cancer patients receiving chemotherapy, although the relationship between TAs and weight loss have been found to be inconsistent. In the present study, significant positive correlations were observed between the “Phantogeusia and Parageusia” and “General Taste Alterations” subscale scores of the CiTAS and recent weight loss (one month before the measurement at T1). Furthermore, the “Decline in Basic Taste,” “Phantogeusia and Parageusia,” and “General Taste Alterations” subscale scores were significantly negatively associated with the percentage of total energy requirements met. All CiTAS subscale scores were also significantly negatively correlated with overall QoL, indicating that greater severity of TAs was associated with poorer nutritional outcomes and reduced QoL.

### Strengths and Limitations

To our knowledge, this study is the first to investigate TAs in conjunction with nutritional intake and QoL in patients receiving ACT across groups differentiated by the number of cycles administered post-surgery. Therefore, focusing on the number of cycles administered may provide a valuable perspective and help to address the existing gap in the literature. In addition, having participants with multiple cancer types instead of a single cancer type is one of the strengths of this study.

This study has a number of limitations that may guide future research designs. The study was single-center, and some of the variables (i.e., daily energy intake and physical activity levels during follow-up) that may affect BMI were not taken into account. Although the sample size is large enough to detect trends within the group of adult patients with cancer, it is not large enough to make firm inferences about the total population of each cancer type. Although the CiTAS and FACT-G are widely utilized and validated instruments, their inherently subjective nature constitutes a limitation. The absence of objective assessments of taste function (e.g., such as direct flavor exposure, gustometry, or taste threshold testing) limits the comprehensiveness of the sensory evaluation. Incorporating objective measures alongside subjective tools may enhance the depth and accuracy of assessment. Although the 3-day dietary recall and 24 h physical activity assessment form are suitable for determining nutrient intake and physical activity level, their inherent susceptibility to recall and reporting bias is a potential limitation. Lack of adjustment for possible confounding factors, such as socioeconomic status or comorbidities, is also a limitation of the study. Furthermore, the outcome data in this study are limited to a 6-month follow-up. The 6-month follow-up period is sufficient to capture short-term trends but limits insights into long-term outcomes. Longer duration prospective studies with larger sample sizes would provide evidence to assess the long-term effects of variables.

## 5. Conclusions

The severity and type of TAs experienced by cancer patients undergoing ACT appear to be influenced by the number of treatment cycles received. A higher number of ACT cycles might be associated with more pronounced TAs, greater nutritional challenges, and reduced QoL, particularly during the early post-treatment period. However, these effects can often be temporary, and both taste function and QoL tend to return to baseline levels following completion of ACT. These findings highlight the importance of systematically monitoring sensory changes and nutritional status in patients receiving multiple cycles of ACT. Supportive interventions—such as ongoing assessment of TAs, individualized nutritional counseling, and taste enhancement strategies—may help mitigate these adverse effects and promote improved nutritional intake and QoL. Further research involving larger sample sizes and extended follow-up durations is warranted to more comprehensively assess the impact of ACT cycle number on TAs and QoL.

## Figures and Tables

**Table 1:** Baseline demographic and clinical characteristics of participants.

Characteristics	Participants		*p*
	<4 ACT Cycle (n = 46)	≥4 ACT Cycle (n = 41)	
**Median age**, years (range)	56.5 (26–71)	60.7 (23–68)	0.254
**BMI (kg/m^2^)** (X ± SD)	25.90 ± 6.85	24.90 ± 8.39	0.465
**Gender**, n (%)			
Female	24 (52.2)	23 (56.1)	0.978
Male	22 (47.8)	18 (43.9)	0.220
**Cancer type**, n (%)			
Breast cancer	12 (26.2)	15 (36.6)	0.960
Lung cancer	9 (19.6)	5 (12.2)	0.452
Stomach cancer	6 (13.0)	8 (19.5)	0.982
Colorectal cancer	11 (23.9)	9 (22.0)	0.957
Pancreatic cancer	6 (13.0)	4 (9.7)	0.726
Lymphoma	2 (4.3)	0 (0.0)	0.024 *
**Chemotherapy regimen**, n (%)			
Anthracycline-based	7	9	0.683
Taxane-based	12	12	1.000
Antracycline and taxane-based	27	20	0.790

Pearson Chi-square, * *p* < 0.05; BMI: Body Mass Index; X: mean; SD: Standard Deviation; ACT: adjuvant chemotherapy.

**Table 2:** Recent weight loss and meeting the energy requirements of participants after the end of treatment (at T1).

	Participants		
	<4 ACT Cycle (n = 46)	≥4 ACT Cycle (n = 41)	*p*
**Recent weight loss, n (%)**			
Moderate (<5%)	34 (73.9)	26 (63.4)	0.044 *
Moderate to severe (5–10%)	7 (15.2)	3 (7.3)	
Severe (>10%)	5 (10.9) **^a^**	12 (29.3) **^b^**	
**Meeting energy requirements, n (%)**			
Hypometabolic (<90% of predicted TEE)	9 (19.5) **^a^**	20 (48.8) **^b^**	0.029 *
Normometabolic (90–110% of predicted TEE)	28 (61.0)	18 (43.9)	
Hypermetabolic (>110% of predicted TEE)	9 (19.5)	3 (7.3)	

Pearson Chi-square, * *p* < 0.05; TEE: Total Energy Expenditure; ACT: adjuvant chemotherapy; different letters in the same row define the significant difference between the groups formed according to the number of ACT received. Bold values are statistically significant (*p* < 0.05). There is no significant difference between genders.

**Table 3:** CiTAS scores at the end of treatment (T1) depend on the demographic and clinical characteristics.

	CiTAS Scores (X ± SD)			
	Decline in Basic Taste	Discomfort	Phantogeusia and Parageusia	General Taste Alterations
**BMI (kg/m^2^)**				
<18	1.00 ± 0.00	1.67 ± 0.00	1.00 ± 0.00	1.00 ± 0.00
18–24.99	1.40 ± 0.75	1.73 ± 0.76	1.55 ± 0.94	1.74 ± 0.93
25–29.99	1.48 ± 0.74	1.79 ± 0.73	1.58 ± 0.90	1.59 ± 0.79
>30	1.51 ± 0.76	1.60 ± 0.60	1.52 ± 1.12	1.43 ± 0.73
	*p* = 0.673	*p* = 0.569	*p* = 0.827	*p* = 0.667
**Age groups (years)**				
18–40	1.54 ± 0.79 ^a&^	1.58 ± 0.50	1.23 ± 0.45	1.55 ± 0.98
41–50	1.37 ± 0.72 ^b&^	1.78 ± 0.83	1.46 ± 0.83	1.71 ± 0.98
51–60	1.40 ± 0.62	1.59 ± 0.68	1.46 ± 0.93	1.46 ± 0.68
61 and over	1.48 ± 0.83	1.91 ± 0.78	1.75 ± 1.02	1.77 ± 0.88
	*p* = 0.894	*p* = 0.433	*p* = 0.311	*p* = 0.798
**Gender**				
Male	1.37 ± 0.65	1.80 ± 0.79	1.50 ± 0.86	1.63 ± 0.78
Female	1.51 ± 0.81	1.72 ± 0.69	1.60 ± 0.97	1.65 ± 0.91
	*p* = 0.632	*p* = 0.901	*p* = 0.357	*p* = 0.704
**Cancer type**				
Breast cancer	1.51 ± 0.85	1.69 ± 0.78	1.64 ± 0.90	1.80 ± 0.97 ^a&^
Lung cancer	1.29 ± 0.62	1.67 ± 0.55	1.10 ± 0.28	1.27 ± 0.68
Stomach cancer	1.40 ± 0.63	1.93 ± 0.83 ^a&^	1.69 ± 1.07	1.66 ± 0.76
Colorectal cancer	1.58 ± 0.82	1.94 ± 0.82 ^a&^	1.87 ± 1.16	1.94 ± 0.85 ^a&^
Pancreatic cancer	1.36 ± 0.67	1.60 ± 0.53	1.20 ± 0.63	1.25 ± 0.63
Lymphoma	1.00 ± 0.00	1.08 ± 0.12 ^b&^	1.33 ± 0.47	1.00 ± 0.00 ^b&^
	*p* = 0.802	*p* = 0.605	*p* = 0.069	*p* = 0.028 *
**Chemotherapy regimen**				
Anthracycline-based	1.31 ± 0.58	1.61 ± 0,65	1.34 ± 0.56	1.40 ± 0.30
Taxane-based	1.46 ± 0.25	1.77 ± 0.67	1.51 ± 0.24	1.61 ± 0.17
Antracycline and taxane-based	1.50 ± 0.44	1.53 ± 0.52	1.64 ± 0.40	1.57 ± 0.05
	*p* = 0.242	*p* = 0.125	*p* = 0.106	*p* = 0.128

Kruskal–Wallis Test, ^&^ one-way ANOVA: post hoc test (Tamhane’s T2). * Bold values are statistically significant (*p* < 0.05). BMI: Body Mass Index. Different letters in the same column define the significant difference between the groups formed according to the number of adjuvant chemotherapies received.

**Table 4:** Quality of life scale scores of participants during treatment and follow-up.

FACT-G	Treatment	Mean (SD)				*η* ^2^
		T0	T1	T2	T3	
Physical well-being	<4 cycle	21.4 (5.2) ^a^	22.8 (4.8)	23.8 (4.4) ^b^	23.6 (4.1) ^b^	**0.256 ^&^**
	≥4 cycle	21.0 (5.4) ^a^	18.1 (5.5) ^b^	20.7 (4.8) ^a^	22.3 (4.5) ^c^	**0.325 ^&^**
		*p* = 0.524	*p* = 0.035 *	*p* = 0.021 *	*p* = 0.085	
			**d = 0.500 ^¥^**	**d = 0.516 ^¥^**		
Social/family well-being	<4 cycle	22.6 (5.6)	21.9 (3.9)	23.2 (4.2)	23.9 (4.6)	
	≥4 cycle	22.2 (4.8) ^a^	19.5 (4.6) ^b^	21.8 (4.1)	22.5 (3.9) ^a^	**0.284 ^&^**
		*p* = 0.225	*p* = 0.046	*p* = 0.057	*p* = 0.087	
Emotional well-being	<4 cycle	16.9 (4.2)	17.4 (5.0)	17.8 (3.6)	18.1 (3.9)	
	≥4 cycle	17.1 (4.9)	17.0 (4.5)	17.5 (3.8)	17.8 (4.2)	
		*p* = 0.076	*p* = 0.150	*p* = 0.236	*p* = 0.145	
Functional well-being	<4 cycle	16.3 (3.7) ^a^	15.8 (3.4)	17.4 (4.1) ^b^	19.2 (3.9) ^c^	**0.305 ^&^**
	≥4 cycle	16.7 (4.1) ^a^	14.5 (3.8) ^b^	15.2 (3.5)	16.1 (3.6) ^a^	**0.228 ^&^**
		*p* = 0.574	*p* = 0.010 *	*p* = 0.024 *	*p* = 0.010 *	
			**d = 0.621 ^¥^**	**d = 0.562 ^¥^**	**d = 0.585 ^¥^**	
Total score	<4 cycle	77.2 (15.6) ^a^	77.9 (12.4)	82.2 (14.6)	84.8 (15.1) ^b^	**0.368 ^&^**
	≥4 cycle	77.0 (13.4) ^a^	69.1 (12.7) ^b^	76.2 (15.0) ^a^	79.7 (14.8) ^a^	**0.320 ^&^**
		*p* = 0.298	*p* = 0.001 *	*p* = 0.028 *	*p* = 0.162	
			**d = 0.590 ^¥^**	**d = 0.500 ^¥^**		

Pearson Chi-square. Different letters in the same row define the significant difference between the follow-up times; *: Repeated measures ANOVA with post hoc Bonferroni. Bold values are statistically significant (*p* < 0.05). FACT-G: Functional Assessment of Cancer Therapy; ACT: adjuvant chemotherapy; SD: Standard Deviation; T0: Baseline; T1: After treatment; T2: 3 months after treatment; T3: 6 months after treatment; d: Cohen’s d effect size (^¥^: medium effects); *η*^2^: Eta-squared effect size (^&^: large effects). There is no significant difference in total and subgroup FACT-G scores between genders at any follow-up time.

**Table 5:** Correlation (at T1) between the taste alteration and quality of life, recent weight loss, and meeting energy requirements.

	CiTAS scores			
	Decline in Basic Taste	Discomfort	Phantogeusia and Parageusia	General Taste Alterations
**Recent weight loss (%BW)**	0.149	0.250	0.166 *	0.262 *
**Meeting energy requirements (%TER)**	−0.248 *	−0.141	−0.130 *	−0.105 *
**FACT-G**				
Physical well-being	−0.357 **	−0.175	−0.173	−0.280 **
Social/family well-being	−0.193	−0.188	−0.157	−0.224 *
Emotional well-being	−0.032	−0.052	−0.015	−0.050
Functional well-being	−0.337 **	−0.294 **	−0.160	−0.287 **
Total score	−0.407 **	−0.240 **	−0.270 **	−0.347 **

Spearman rho test, bold values are statistically significant (* *p* < 0.05, ** *p* < 0.01), BW: body weight, TER: Total Energy Requirement.

## Data Availability

The data presented in this study are available on request from the corresponding author due to personal data protection.

## Abbreviations

The following abbreviations are used in this manuscript:

ACT	Adjuvant chemotherapy
TAs	Taste alterations
QoL	Quality of life
BeBis	Nutrition Information System
CiTAS	Chemotherapy-induced Taste Alteration Scale
FACT-G	Functional Assessment of Cancer Therapy
PWB	Phsical well-being
SWB	Social and family well-being
EWB	Emotional well-being
FWB	Functional well-being
BMR	Basal metabolic rate
PAL	Physical activity level
